# Long-Term Effects of High-Intensity Interval Training (HIIT) on Cardiac Function and Mortality in Heart Failure

**DOI:** 10.7759/cureus.91972

**Published:** 2025-09-10

**Authors:** Mikhaya Seby, Sarath Vayolipoyil, Maria Akbar, Adi Ahmed, Mohamed A.M.Imam, Samraiz Nafees, Nisarg Shah, Asya Azad, Muhammad Aashan

**Affiliations:** 1 Acute Medicine, York and Scarborough Teaching Hospitals National Health Service (NHS) Foundation Trust, Scarborough, GBR; 2 General Medicine, York and Scarborough Teaching Hospitals National Health Service (NHS) Foundation Trust, Scarborough, GBR; 3 Internal Medicine, York and Scarborough Teaching Hospitals National Health Service (NHS) Foundation Trust, Scarborough, GBR; 4 Emergency Medicine, York and Scarborough Teaching Hospitals National Health Service (NHS) Foundation Trust, Scarborough, GBR; 5 General Internal Medicine, Tameside Hospital, Manchester, GBR; 6 Internal Medicine, Pakistan Institute of Medical Sciences, Islamabad, PAK

**Keywords:** cardiac function, heart failure, high-intensity interval training, quality of life, symptom severity

## Abstract

Introduction: Heart failure (HF) is one of the causes of morbidity and mortality. High-intensity interval training (HIIT) has been suggested as a positive intervention that can be practiced to help optimize heart function and overall health. However, the effect of HIIT in the long-term on heart functioning, the level of symptoms shown by patients with HF, and the survival of the affected people are poorly understood. This study explores the effects of a long-term HIIT intervention on individuals who have HF.

Methods: This was a longitudinal observational study conducted in the cardiac department of Pakistan Institute of Medical Sciences (PIMS) in Islamabad, Pakistan, from July 2024 to January 2025. Purposive non-probability sampling was used to recruit all HF patients, with 158 patients aged 30 years and older participating. The demographic information and research instruments used at baseline, three months, and six months include the Kansas City Cardiomyopathy Questionnaire (KCCQ-12), Duke Activity Status Index (DASI) questionnaires, and New York Heart Association (NYHA) classification. IBM SPSS version 26 (IBM Corp., Armonk, NY, US) was used to conduct statistical tests, including repeated-measures ANOVA, Pearson correlation, and multiple linear regression.

Results: The sample size consisted of 158 participants (N = 113, 71.0% men; N = 45, 29.0% women), with a mean age of 56.3 ± 12.4 years. There was a significant improvement in both KCCQ-12 and DASI (p < 0.01), whereas the NYHA classification worsened (p < 0.001). Increased levels of HIIT were associated with improved functional capacity and quality of life (r = 0.392, p < 0.01 for DASI; r = 0.215, p < 0.01 for KCCQ-12) and reduced symptom severity (r = -0.265, p < 0.01 for NYHA). The main predictors of these outcomes identified through regression analysis included HIIT, age, gender, and comorbidities. Gender differences were demonstrated by women reporting better outcomes and men exhibiting worse indicators concerning symptoms.

Conclusion: Long-term HIIT is highly effective in improving cardiac performance, decreasing the severity of HF symptoms, and improving the quality of life among HF patients. These findings demonstrate the promising potential of HIIT as an effective adjunctive treatment in managing HF, as it can bring significant benefits to patients of diverse ages and backgrounds. Early HIIT, tailored to the individual needs of each patient, has the potential to improve clinical outcomes and increase survival rates in HF populations.

## Introduction

Heart failure (HF) is rapidly becoming a worldwide health problem, and the prevalence is on the rise because of an aging population, increased survival, and the number of risk factors, especially in high-income economies. HF is a significant health burden affecting five million or more people in the United States, and trends have shown that despite treatment breakthroughs, it has a 50% five-year mortality rate [1,2]. Physical activity is essential in the prevention of many chronic diseases, and new studies indicate that high-intensity interval training (HIIT) is more effective at optimizing health benefits compared to moderate-intensity physical activity [3].

HIIT involves repeated sessions of intense exercise followed by recovery, which is used to improve sports performance and overall health in sedentary individuals. Despite its extensive use, there are no standard definitions of HIIT intensity, and additional studies are needed to optimize HIIT protocols and improve performance and clinical outcomes [4]. Moreover, HIIT has demonstrated potential improvements in patients living with chronic illnesses such as cardiovascular and pulmonary diseases. Due to its alternating high- and low-intensity phases, it allows for a higher workload and an enhancement of functional capacity compared to moderate-intensity continuous training (MICT) [5].

HIIT is an effective technique for increasing VO_2_ max and endurance, applicable to all individuals, including athletes. It exhibits a high intensity that stimulates molecular pathways, promoting mitochondrial biogenesis and angiogenesis, even during shorter-duration exercise [6]. In active young adults, HIIT performed in the short term was reported to have a significant effect on increasing VO_2_ max, oxygen pulse, and power output [7].

A systematic review of 65 studies revealed that short- and long-term HIIT profoundly enhanced VO_2_ max and some of the cardiometabolic indicators, including blood pressure, waist circumference, and fasting glucose, among overweight and obese adults. HIIT had an insignificant effect on normal-weight populations, except for increasing VO_2_ max [8]. Additionally, HIIT has been shown to have similar or better effects than MICT in cardiac rehabilitation with a low rate of adverse events. A meta-analysis found that HIIT, compared to MICT, significantly increased cardiorespiratory fitness (VO_2_ peak) in patients with lifestyle-related chronic diseases, with an absolute difference of 9.1% [9,10].

Rationale

HF is a condition that affects various aspects of day-to-day life, making physical activity difficult and recovery challenging. Exercise is often encouraged as a healthy habit to maintain a healthy heart. However, not every type of exercise yields the same results, particularly when it comes to enhancing the heart's long-term efficiency. The focus is increasingly on the possibility that there may be further or more lasting benefits to other forms of exercise, such as HIIT.

Although some studies indicate that HIIT is efficacious in improving exercise capacity and specific markers related to heart function, there is limited information regarding its long-term role. Specifically, it is unclear whether the effects of HIIT are cumulative and whether the intervention could result in a feasible prescription for mortality reduction in patients with HF. This research will address that gap by focusing on the long-term impact of HIIT on heart health and survival outcomes. These findings can be utilized to develop an exercise prescription that is more effective in improving the lives of patients with cardiac failure.

Primary objective

The primary objective is to determine the cardiovascular effects of HIIT on the long-term outcomes of patients with HF.

Secondary objectives

The secondary objectives are to evaluate the effects of long-term HIIT on all-cause and cardiovascular or heart-related deaths among patients with HF, to evaluate changes in symptom burden and health-related quality of life (QoL), and to investigate the safety, compliance, and practicality of long-term HIIT in a HF population.

## Materials and methods

Research design

The method used in this research study is a longitudinal observational research study to examine the effect of HIIT over time, in terms of its impact on cardiac functional capacity and mortality among patients with HF. Data were assessed at three time points (baseline, three months, and six months). Demographics and HIIT participation were evaluated only at baseline; however, patient-reported outcome measures, such as functional status and QoL responses, were assessed at the three subsequent visits. The methodology enabled the tracking of modifications that occur in people over time and evaluated the long-term effects of HIIT. A control group (such as MICT or standard care) was not included because all enrolled participants were already receiving structured HIIT as part of the rehabilitation program. Withholding exercise would have been considered substandard care in this setting. Therefore, the study was designed to assess within-subject changes over time rather than to establish direct causal comparisons between exercise modalities. The Institutional Review Board of Pakistan Institute of Medical Sciences, Islamabad, Pakistan, approved the study under protocol number PIMS/ERB/24/33. All participants provided informed consent, as the objectives and procedures of the survey were clearly explained to them. The involvement was voluntary, and the patients were promised that they could withdraw at any point without any implications to their treatment.

Study setting and duration

The study was conducted at the cardiac department of PIMS in Islamabad, Pakistan. Data were collected over a six-month period, from July 2024 to January 2025. The purposive non-probability sampling method was used to recruit participants.

Study cohort

The sample size was calculated using the WHO formula to determine the sample proportion (conservative proportion). The proportion in the population of interest is 0.90, the margin of error is 0.05, and the confidence interval is 0.95 [11]. The calculated sample size was approximately 300 participants, based on the above parameters, to achieve the best statistical power and representativeness. Nevertheless, the actual circumstances in the real world (limited time, availability of participants, and their compliance with follow-up) reduced the initial number of recruited people to 200. The final group, comprising 158 participants, was established after considering the inclusion and exclusion criteria, as well as the rate of loss to follow-up.

Although the final sample size is limited, the study is internally valid due to its longitudinal design with repeated measurements, which provides valuable information about the long-term outcomes of HIIT in patients with HF. However, the reduced sample size compared to the initial calculation limits the external validity of the findings and may restrict their generalizability, particularly to more diverse populations or healthcare settings.

Inclusion criteria

The inclusion criteria were a confirmed diagnosis of HF, age of 30 years and above, and medical clearance to enroll in a HIIT program. The age of 30 was selected because it represents an age group where some cardiovascular risk factors, such as high blood pressure and diabetes, have increased, potentially affecting the heart's functioning. Even though younger people may have the potential to develop HF, cardiac changes and comorbidities are more common in people older than 30, which makes this category of patients an applicable target of such interventions as HIIT. All participants gave written informed consent, and only those willing and capable of keeping follow-up measurements at three and six months were selected.

Exclusion criteria

Exclusion criteria were the presence of severe musculoskeletal or neurological conditions that may prevent physical activity, recent acute cardiac events in the previous four weeks, or a significant comorbid illness that may otherwise jeopardize safety during exercise. People with cognitive disability or who could not commit or were reluctant to follow the follow-up plan were also eliminated.

Data collection tools

This study employed a structured questionnaire (five domains) that included demographic information, participation in HIIT, and three standardized tools to measure the level of HF severity, the level of functioning, and QoL. All the tools were used in their native English format because none of them had been translated into the local language at the time of the investigation. We were offered help where necessary to ensure we understood and provided correct answers. The data were measured three times at baseline, three months, and six months, except for demographic and HIIT specifics, which were only measured at baseline.

Demographic information

The initial part of the questionnaire collected baseline demographic and clinical information. These were age, gender, education level, marital status, and employment status. Moreover, the participants were requested to describe how long they had been diagnosed with HF and, in case they knew it, what kind of HF they had been diagnosed with. Clinical information also included whether the participants had comorbid medical issues (hypertension, diabetes, or kidney disease) or were at the moment taking any medications to manage HF. It was crucial to describe the sample and examine potential subgroup differences in terms of demographic and clinical variations.

HIIT participation

A self-structured screening instrument specifically designed for this study was used to assess participation in HIIT. This was not intended for diagnostic purposes, but rather to capture some general trends in how participants participate in HIIT. The instrument consisted of four questions that measured the number of HIIT sessions per week, the average session duration, the years of regular HIIT participation, and the level of perceived HIIT intensity. All answers ranged from 1 to 4, with higher scores indicating that more people participated in extracurricular activities. Its total score, ranging from 4 to 16, was analyzed as a continuous variable. The participants were divided into three groups based on their total score, classified as low participation (3-5), moderate participation (6-8), and high participation (9-16). It should be noted that the study did not prescribe or standardize a specific HIIT protocol. Instead, participation was captured through a self-structured questionnaire based on patient self-reports regarding session frequency, duration, years of participation, and perceived intensity.

Kansas City Cardiomyopathy Questionnaire

The Kansas City Cardiomyopathy Questionnaire (KCCQ-12) was initially developed in 2000 by Spertus and Jones at the Mid-America Heart Institute and measures four variables: frequency of limitation, QoL, frequency of symptoms, and social limitation. The responses on all items are counted and translated to a 0-100 scale, where bigger numbers denote a higher health status. A cumulative domain score is calculated by averaging domain scores. The KCCQ-12 is a relatively short, self-administered scale, and good evidence of internal consistency is provided, indicating the scale has high internal consistency, with Cronbach's alpha of 0.89. The following study utilized this effective instrument to assess how the symptoms of HF impacted the lives of patients involved and their overall condition [12]. The KCCQ-12 scale was employed under a limited license agreement (License ID #13565) granted by Outcomes Instruments, LLC, authorizing its use for the present study.

Duke Activity Status Index

The Duke Activity Status Index (DASI) is a self-directed questionnaire developed by Hlatky et al. in 1989 to assess an individual's functional capacity, particularly in cardiovascular endurance. It assesses the likelihood of participating in a body of all physical activities that have a weighted level, which is then assigned to various activities based on their metabolic equivalent (MET) value. There are 12 items in DASI, all of which are associated with activities of daily living, such as walking indoors, climbing stairs, or performing light housework/recreational sports. If the participant reports being able to perform the activity, the corresponding weight is added to the total score; if not, a zero is recorded. The overall score ranges from 0 to 58.2, with a higher score indicating better functional capacity. The scale has demonstrated adequate internal consistency, with Cronbach's alpha score of approximately 0.86, which is a good measure of test strength. It has been used in this paper to evaluate the physical functioning and exercise tolerance of participants with HF [13]. The DASI was licensed for non-commercial academic research use under a formal agreement with Greenlight Health Data Solutions, Inc., the authorized licensing body on behalf of Duke University.

New York Heart Association functional classification

In this study, the New York Heart Association (NYHA) functional classification was used. This classification categorized the physical restrictions that participants experienced based on their symptoms of HF. People are classified into four classes (I-IV) based on the impact of fatigue or shortness of breath on their physical activity. Class I suggests that there is no limitation in the capacity to perform daily physical activities, while Class IV indicates that severe symptoms are present at rest. The NYHA system, introduced in 1928, remains one of the most widely used and straightforward methods for assessing functional status in patients with HF. Although it is not applied numerically and may be evaluated by both clinicians and through self-report based on standard criteria, the level of reliability of the classification system has been proven sufficient, despite its subjectivity [14]. Formal permission to reproduce the American Heart Association copyrighted material was obtained through a copyright use agreement (Invoice #21377-M).

Procedure

Once they had given their informed consent, participants were invited to participate in the study. The standardized questionnaire, which contained both standard and researcher-made questions, was primarily administered in the self-administration mode. Nevertheless, there were trained data collectors to help those who needed assistance because of reading problems or an inability to understand something. To ensure consistency, the data collectors were standardizedly trained and conducted under identical administration protocols. It was provided non-directively to minimize the interviewer's influence and maintain neutrality. The answers were also evaluated anonymously, and any identifying information was removed before submission to maintain confidentiality. This was achieved through respect, accessibility, and cultural appreciation, allowing for the involvement of people with diverse educational levels, health conditions, and backgrounds.

Statistical analysis

The data analysis was conducted using IBM SPSS Statistics 26 (IBM Corp., Armonk, NY, US). The demographic information on the participants was summarized using descriptive statistics, including mean, standard deviations, frequency, and percentage. Kolmogorov-Smirnov and Shapiro-Wilk tests were used to assess the normality of the outcome variables, which included HIIT scores, KCCQ scores, NYHA class, and DASI. To investigate the linear relationship between exercise level, symptom severity, physical functioning, and QoL, Pearson correlation analysis was performed. A comparison of gender on functional and clinical outcomes at baseline was conducted using independent sample t-tests, with the magnitude of the group differences reported in terms of Cohen's d effect sizes. Repeated-measures ANOVA was used to assess within-subject changes over the three distributions to three time points (T1, T2, and T3) using the KCCQ, NYHA, and DASI scores.

Additionally, a mixed factorial repeated-measures ANOVA was used to investigate the interaction effects of HF duration and time on these outcomes. Estimates of effect sizes were reported as partial η². Multiple linear regression was also applied to prospectively investigate the variation in KCCQ, NYHA, and DASI scores over time. The independent variables included HIIT total score, age, gender, type of HF, and comorbid conditions. The standardized beta coefficients of these models were also provided to measure the influence of the predictors.

Lastly, chi-squared tests were performed to assess relationships between categorical variables, such as the type and duration of HF onset, and the presence of comorbid medical conditions. Analysis of all the statistics was two-sided, and the level of significance adopted was p < 0.05.

Ethical guidelines

This research was conducted in accordance with established ethical standards for the study of human participants. Extra attention was paid to the rights and safety of people with chronic health conditions because the risks of receiving physical and emotional injuries were avoided. Recruitment was done with the least amount of coercion and undue pressure. The participants were assured that their clinical care would not be affected by their decision to take part or not. The undertakings were conducted with dignity, sensitivity to culture, and a commitment to safeguard the dignity and autonomy of every participant.

## Results

Table 1 shows the demographic and clinical data of the respondents (N = 158). The majority of participants were aged 60 years and above (N = 66, 42%), followed by the second largest group, those aged 50-59 years (N = 34, 22%). The sample consisted predominantly of men (N = 113, 71%) and included a large number of respondents who were married (N = 61, 39%) or single (N = 42, 27%). In terms of education, the majority (N = 57, 36%) completed primary education only, while fewer had a bachelor's degree (N = 8, 5%) or higher qualifications. Employment status indicated that they were either unemployed (N = 53, 34%) or retired (N = 51, 32%). The diagnosis of HF ranged most frequently between six months and one year (N = 54, 34%); 69 (44%) reported preserved ejection fraction (HFpEF), 40 (25%) reported reduced ejection fraction (HFrEF), and 49 (31%) did not know their type. Of the comorbidities, hypertension (N = 52, 33%) and diabetes (N = 41, 26%) were the most commonly reported. The vast majority of individuals were already receiving HF medications (N = 112, 71%).

**Table 1:** Demographic details of participants (N = 158) N: number of participants; f: frequency; %: percentage.

Variable	f	%
Age	-	-
30-39 years	26	16
40-49 years	32	20
50-59 years	34	22
60 years and above	66	42
Gender	-	-
Male	113	71
Female	45	29
Marital status	-	-
Single	42	27
Married	61	39
Widowed	37	23
Divorced	18	11
Educational level	-	-
No formal education	30	19
Primary	57	36
Secondary	44	28
Intermediate	17	11
Bachelor's degree	8	5
Master's degree	2	1
Employment status	-	-
Student	27	17
Employed	27	17
Unemployed	53	34
Retired	51	32
Duration of heart failure diagnosis	-	-
Less than 6 months	40	25
6 months-1 year	54	34
1-3 years	44	28
More than 3 years	20	13
Type of heart failure (if known)	-	-
HFrEF (reduced ejection fraction)	40	25
HFpEF (preserved ejection fraction)	69	44
Do not know	49	31
Do you have any of the following medical conditions?	-	-
Diabetes	41	26
Hypertension	52	33
Chronic kidney disease	36	23
Previous heart attack	18	11
Obesity	6	4
None of these	5	3
Are you currently taking any heart failure medications?	-	-
Yes	112	71
No	46	29

Table 2 presents the results of normality testing for functional and clinical outcome measures, using the Kolmogorov-Smirnov and Shapiro-Wilk tests (N = 158). In both tests, all variables had non-significant p-values (p > 0.05), indicating that the data were normally distributed. In particular, considering HIIT, the results of the Shapiro-Wilk and Kolmogorov-Smirnov tests indicated p-values of 0.072 and 0.113, respectively. The KCCQ results were p = 0.454 and p = 0.200, respectively. The classification carried out by the NYHA revealed p-values of 0.081 and 0.089, whereas the DASI revealed p-values of 0.063 and 0.127. All p-values were greater than 0.05; thus, parametric tests were deemed suitable to pursue.

**Table 2:** Tests of normality for functional and clinical outcome measures using Kolmogorov–Smirnov and Shapiro–Wilk tests (N = 158) All variables met normality criteria (p > 0.05); df: degrees of freedom, parametric analyses deemed appropriate.

Variables	Kolmogorov-Smirnov			Shapiro-Wilk		
	Statistic	df	p	Statistic	df	p
High-intensity interval training (HIIT)	0.077	158	0.113	0.983	158	0.072
Kansas City Cardiomyopathy Questionnaire (KCCQ)	0.058	158	0.200	0.991	158	0.454
New York Heart Association (NYHA)	0.081	158	0.089	0.979	158	0.081
Duke Activity Status Index (DASI)	0.073	158	0.127	0.985	158	0.063

Table 3 presents the Pearson correlation coefficients between HIIT and symptom severity (NYHA), functional capacity (DASI), and QoL (KCCQ) in N = 158 participants. HIIT was moderately and positively related to DASI (r = 0.392, p < 0.01) and KCCQ (r = 0.215, p < 0.01) in that the more a person engaged in HIIT workouts, the higher the functional capacity and QoL, respectively. HIIT and NYHA class showed an inverse relationship (r = 0.265, p < 0.01), indicating that greater degrees of exercise were associated with milder symptoms (since NYHA is reverse-coded). On the same note, KCCQ was positively correlated with DASI (r = 0.176, p < 0.05) and negatively correlated with NYHA (r = -0.301, p < 0.01), indicating that a higher QoL was also associated with a higher level of function and a lesser degree of symptom severity. Finally, NYHA was significantly negatively correlated with DASI (r = -318, p < 0.01), which means that more severe symptoms were linked with a lower level of functional capacity.

**Table 3:** Pearson correlation matrix for exercise, symptom severity, functional capacity, and quality of life (N = 158) *p < 0.05, **p < 0.01 considered significant. Positive correlations indicate direct relationships, and negative correlations indicate inverse relationships. NYHA is reverse-coded (higher = worse function), while higher scores on HIIT, KCCQ, and DASI represent better function or quality of life.

Variable	High-intensity interval training (HIIT)	Kansas City Cardiomyopathy Questionnaire (KCCQ)	New York Heart Association (NYHA) class	Duke Activity Status Index (DASI)
High-intensity interval training (HIIT)	-	0.215^**^	–0.265^**^	0.392^**^
Kansas City Cardiomyopathy Questionnaire (KCCQ)	-	-	–0.301^**^	0.176^*^
New York Heart Association (NYHA) class	-	-	-	–0.318^**^
Duke Activity Status Index (DASI)	-	-	-	-

Table 4 presents strong features of gender differences with all measured variables. Women scored better on HIIT (M = 10.02 vs. 9.15), KCCQ QoL (M = 29.65 vs. 27.92), and DASI functional capacity (M = 16.48 vs. 15.12) than men (p = 0.003). Men, on the other hand, had greater scores concerning NYHA classes (M = 2.68 vs. 2.31), but worse HF condition (p < 0.001). The effect size values were moderate to large (Cohen's d = 0.56-0.85), reflecting the presence of decent gender-related differences in clinical and functional parameters.

**Table 4:** Gender differences in HIIT, KCCQ, NYHA, and DASI scores based on independent sample t-tests N: number of participants; M: mean; SD: standard deviation; LL: lower limit; UL: upper limit; CI: confidence interval; independent t-test; p < 0.01 (two-tailed) was considered statistically significant and is denoted with double asterisks (**).

Variable	Male (N = 113); M ± SD	Female (N = 45); M ± SD	t	p	95% CI LL	95% CI UL	Cohen’s d
High-intensity interval training (HIIT)	9.15 ± 1.50	10.02 ± 1.38	–3.03	0.003^**^	–1.44	–0.30	0.59
Kansas City Cardiomyopathy Questionnaire (KCCQ)	27.92 ± 3.21	29.65 ± 2.80	–3.01	0.003^**^	–2.87	–0.59	0.56
New York Heart Association (NYHA) class	2.68 ± 0.49	2.31 ± 0.52	4.17	<0.001^**^	0.20	0.54	0.74
Duke Activity Status Index (DASI)	15.12 ± 1.66	16.48 ± 1.43	–4.37	<0.001^**^	–1.98	–0.74	0.85

Table 5 summarizes the functional status, symptom burden, and physical capacity of patients with HF (N = 158) at three points in time. KCCQ-12 was significantly higher as reflecting an improvement over time T1 (M = 28.84, SD = 3.63) at T3 (M = 30.22, SD = 3.89), F(2, 314) = 6.64, p = 0.002, where partial η² indicated a small-to-moderate effect, that is, 0.041. Equally, NYHA functional class scores grew significantly between T1 (M = 2.49) and T3 (M = 2.82), F(2, 314) = 13.52, p < 0.001, partial η² = 0.079, representing a moderate effect. DASI, which assesses physical capacity, also enhanced T1 (M = 15.42) to T3 (M = 16.74), F(2, 314) = 11.12, p < 0.001, with partial η² = 0.066. All of these findings, considered separately, suggest a significant improvement in self-reported health status, functional classification, and ability to perform physical activity in patients over time.

**Table 5:** Means (M), standard deviations (SD), and repeated-measures ANOVA results for functional status, symptom burden, and physical capacity across three time points (N = 158) SS: sum of squares; MS: mean square; df: degrees of freedom; F: F-ratio. Repeated-measures ANOVA was conducted to assess changes across three time points: T1: baseline; T2: 3 months; T3: 6 months. Results show significant improvements in KCCQ-12 (p = 0.002), NYHA (p < 0.001), and DASI (p < 0.001) scores, indicating enhanced heart failure-related health status, functional class, and physical capacity over time. Partial eta squared (η²ₚ) reflects small-to-moderate effect sizes; **p < 0.01 considered significant.

Variable	Time point	N	M ± SD	SS (time)	MS (time)	df	p	F	Partial η^2^
Kansas City Cardiomyopathy Questionnaire (KCCQ)	T1	158	28.84 ± 3.63	-	-	-	-	-	-
-	T2	158	30.18 ± 3.45	-	-	-	-	-	-
-	T3	158	30.22 ± 3.89	193.35	96.67	2	0.002^**^	6.64	0.041
New York Heart Association (NYHA) class	T1	158	2.49 ± 0.58	-	-	-	-	-	-
-	T2	158	2.66 ± 0.57	-	-	-	-	-	-
-	T3	158	2.82 ± 0.61	8.56	4.28	2	<0.001^**^	13.52	0.079
Duke Activity Status Index (DASI)	T1	158	15.42 ± 1.92	-	-	-	-	-	-
-	T2	158	16.12 ± 1.85	-	-	-	-	-	-
-	T3	158	16.74 ± 1.87	66.421	33.210	2	<0.001^**^	11.12	0.066

Table 6 demonstrates that KCCQ and DASI scores improved over time across the HF duration groups (e.g., <6 months: KCCQ improved 29.45 to 33.20; DASI improved 15.82 to 17.45). Conversely, the NYHA scores were trending upward, reporting adverse changes in functional class (<6 months: 2.45-2.95). The trends across short-duration and long-duration groups were similar, with an increase in symptom burden (NYHA class) with time despite improved QoL and functional capacity.

**Table 6:** Repeated-measures ANOVA showing the effect of heart failure duration on health outcomes over time (KCCQ, NYHA, and DASI) N: number of participants within each heart failure duration group; M: mean; SD: standard deviation; KCCQ: Kansas City Cardiomyopathy Questionnaire; NYHA: New York Heart Association; DASI: Duke Activity Status Index. Repeated-measures ANOVA was conducted to assess changes across three time points: T1: baseline; T2: 3 months; T3: 6 months.

Duration of diagnosis	N	KCCQ-time 1; M ± SD	KCCQ-time 2; M ± SD	KCCQ-time 3; M ± SD	NYHA-time 1; M ± SD	NYHA-time 2; M ± SD	NYHA-time 3; M ± SD	DASI-time 1; M ± SD	DASI-time 2; M ± SD	DASI-time 3; M ± SD
Less than 6 months	40	29.45 ± 3.30	32.10 ± 2.90	33.20 ± 2.95	2.45 ± 0.60	2.60 ± 0.55	2.95 ± 0.55	15.82 ± 2.11	16.84 ± 1.85	17.45 ± 1.65
6 months–1 year	54	28.76 ± 3.75	30.75 ± 3.10	32.00 ± 3.40	2.57 ± 0.50	2.67 ± 0.51	2.90 ± 0.57	15.94 ± 1.89	16.73 ± 1.71	17.29 ± 1.72
1–3 years	44	28.45 ± 3.92	30.80 ± 3.00	31.90 ± 3.50	2.55 ± 0.63	2.68 ± 0.64	2.85 ± 0.62	15.48 ± 1.89	16.52 ± 1.81	17.21 ± 1.68
More than 3 years	20	28.70 ± 3.40	30.60 ± 3.20	31.85 ± 3.60	2.20 ± 0.62	2.70 ± 0.66	2.90 ± 0.75	16.05 ± 1.91	16.78 ± 1.66	17.40 ± 1.51

Table 7 indicates that time was an influential factor on all the outcomes. KCCQ scores were improved over time points (F(2, 308) = 8.94, p < 0.001, η^2^ = 0.055), whereas the NYHA class was worse (F(2, 308) = 20.82, p < 0.001, 0.116), and DASI scores were higher (F(2, 308) = 15.62, p < 0.001, 0.165). The significant time × duration interactions were also noted in KCCQ (p = 0.027), NYHA (p = 0.001), and DASI (p = 0.003), indicating that the change in QoL, functional class, and physical capacity enjoyed different-duration HF.

**Table 7:** Repeated-measures ANOVA of time and time × duration effects on health outcomes (N = 158) SS: sum of squares; MS: mean square; df: degrees of freedom; F: F-ratio; η²: effect size. *p < 0.05; **p < 0.01 considered significant.

Outcome	Effect	SS	MS	F(df)	p	Partial η²
Kansas City Cardiomyopathy Questionnaire (ΔKCCQ)	Time	220.35	110.18	8.94(2, 308)	<0.001^**^	0.055
-	Time × duration	134.22	22.37	2.44(6, 308)	0.027^*^	0.030
New York Heart Association class (ΔNYHA)	Time	13.750	6.875	20.82(2, 308)	<0.001^**^	0.116
-	Time × duration	7.860	1.310	4.00(6, 308)	0.001^**^	0.072
Duke Activity Status Index (ΔDASI)	Time	145.62	72.81	15.62(2, 308)	<0.001^**^	0.165
-	Time × duration	64.48	10.75	3.41(6, 308)	0.003^**^	0.097

Table 8 shows that multiple variables significantly predicted changes in KCCQ, NYHA class, and DASI scores, with HIIT, age, gender, type of HF, and comorbid conditions being part of this multiplicity. In KCCQ, age was inversely correlated to improvement, and HIIT was also correlated inversely, but gender was positively correlated with improvement, which means that more improvements would be witnessed in the female group. Age, HIIT, and gender were also negatively associated with the NYHA classification, but age was the strongest predictor, which means that older people have less improvement. The nature of the HF and comorbidity were also high predictors, and the presence of the comorbidities had negative impacts on the KCCQ and DASI scores, but positive impacts on the changes in the severity of NYHA. By the same measure, in the DASI, HIIT and age contributed negatively, though it implies that women improved more compared to men. The findings of the present work highlight the fact that the interconnections between physical activity (HIIT), age, gender, and other clinical variables are complicated when considering the evolution of HF and its related outcomes.

**Table 8:** Multiple linear regression analyses predicting changes in KCCQ, NYHA class, and DASI scores from baseline to follow-up This table presents the results of multiple linear regression analyses predicting changes in Kansas City Cardiomyopathy Questionnaire (ΔKCCQ), New York Heart Association (ΔNYHA) classification, and Duke Activity Status Index (ΔDASI) scores over time. Independent variables included high-intensity interval training (HIIT) total score, age, gender, type of heart failure, and presence of comorbid medical conditions. p-values less than 0.05 indicate statistical significance. CI: confidence interval; LL: lower limit; UL: upper limit; SE: standard error.

Outcome variable	Predictor	B	95% CI LL	95% CI UL	SE	β	p
ΔKCCQ	(Constant)	2.498	1.107	3.889	0.707	-	0.001^**^
^-^	HIIT total	-0.320	-0.506	-0.134	0.094	-0.165	0.001^**^
^-^	Age	-0.904	-1.275	-0.533	0.188	-0.248	<0.001^**^
^-^	Gender	1.245	0.609	1.881	0.322	0.189	<0.001^**^
^-^	Heart failure type	-0.498	-0.806	-0.190	0.156	-0.143	0.002^**^
^-^	Comorbid condition	-0.329	-0.528	-0.130	0.101	-0.123	0.001^**^
ΔNYHA	(Constant)	1.206	0.580	1.832	0.318	-	<0.001^**^
^-^	HIIT total	-0.088	-0.136	-0.040	0.024	-0.212	<0.001^**^
^-^	Age	-0.237	-0.321	-0.153	0.043	-0.316	<0.001^**^
^-^	Gender	-0.214	-0.354	-0.074	0.071	-0.170	0.003^**^
^-^	Heart failure type	0.103	0.032	0.174	0.036	0.145	0.005^**^
^-^	Comorbid condition	0.092	0.037	0.147	0.028	0.135	0.001^**^
ΔDASI	(Constant)	3.191	1.614	4.768	0.793	-	<0.001^**^
^-^	HIIT total	-0.255	-0.376	-0.134	0.061	-0.242	<0.001^**^
^-^	Age	-0.144	-0.264	-0.024	0.061	-0.123	0.019^*^
^-^	Gender	-0.532	-0.865	-0.199	0.169	-0.183	0.002^**^
^-^	Heart failure type	0.204	0.043	0.365	0.082	0.148	0.014^**^
^-^	Comorbid condition	-0.187	-0.302	-0.072	0.058	-0.152	0.002^**^

Table 9 indicates that the type of HF is significantly associated with the length of time from diagnosis (χ^2^(6) = 22.8, p = 0.001). In HFrEF, most patients had been diagnosed within the last six months, and in HFpEF, more patients were diagnosed between six months and three years prior. A significant number of participants did not realize their particular type of HF, and the majority of them also belonged to the early stages of HF (<1 year). This indicates that patients with HFpEF have a more extended history of HF than those with HFrEF and shows inadequacies in the realization of the diagnosis.

**Table 9:** Descriptive statistics of demographic variables (type of heart failure and duration of heart failure diagnosis) f: frequency; %: percentage; df: degree of freedom; x^2^: effect size; p: level of significance; p-values calculated using the chi-squared test; **p < 0.01 considered significant.

Type of heart failure	Less than 6 months	6 months-1 year	1-3 years	More than 3 years	Total	p	x^2^(df = 6)
HFrEF (reduced ejection fraction)	22	8	6	4	40	-	-
HFpEF (preserved ejection fraction)	8	26	24	11	69	-	-
Don't know	10	20	14	5	49	-	-
Total	40	54	44	20	158	0.001^**^	22.8

Table 10 indicates a significant association between the type of HF and the presence of comorbid conditions (χ²(10) = 22.7, p = 0.012). Hypertension and diabetes were the most frequently reported conditions, with both being more common among patients with HFpEF. Chronic kidney disease and previous heart attack were also observed, with relatively higher counts in the HFpEF group. A small number of participants (n = 10, 6.3%) reported having no comorbidities. These results suggest that HFpEF patients tend to have a higher burden of medical comorbidities compared to those with HFrEF.

**Table 10:** Descriptive statistics of demographic variables (type of heart failure and any medical condition) f: frequency; %: percentage; df: degree of freedom; x^2^: effect size; p: level of significance; p-values calculated using the x^2^ test. Comorbid conditions are not mutually exclusive; participants may have reported more than one condition. *p < 0.05; **p < 0.01 considered significant.

Type of heart failure	Diabetes	Hypertension	Chronic kidney disease	Previous heart attack	Obesity	None of these	Total	p	x^2^(df = 10)
HFrEF (reduced ejection fraction)	14	13	6	3	3	1	40	-	-
HFpEF (preserved ejection fraction)	17	22	13	7	5	5	69	-	-
Don't know	11	14	10	6	4	4	49	-	-
Total	42	49	29	16	12	10	158	0.012^*^	22.7

## Discussion

This research provides strong evidence that HIIT can lead to improvements in cardiac function, QoL, and symptom burden in individuals with HF when used as a long-term intervention. QoL has a positive association with HIIT participation in our research. The finding aligns with other research findings that inactive adults engaged in HIIT interventions felt a dramatic improvement in physical and mental QoL, mostly when training was regularly cost-effective [15]. Furthermore, in our study, a higher amount of HIIT was strongly associated with a reduction in NYHA class, indicating an improvement in the symptom characteristics of HF patients. This finding is consistent with existing evidence that HIIT is complementary to the benefits of improving cardiac functioning and treating cardiac symptoms in multimodal cardiac rehabilitation programs [16]. Our findings reveal a statistically significant, moderate positive correlation between engagement in HIIT and functional capacity. This is supported by clinical evidence that HIIT induces significantly greater increases in VO_2_ peak and walking distance, with no additional cardiovascular risk, compared to moderate continuous training [17]. Among patients with a lower NYHA score, we observed an improved QoL in our analysis. This finding is consistent with the results of other studies, which have also demonstrated that symptom severity and the likely health status rating of patients with varying symptom severity levels are linked in such a way that those with lower levels have a more positive outlook on their health status [18]. Our study has shown a weak positive correlation between the functional capacity and QoL. This result aligns with other research findings, which suggest that increased physical independence and mobility are associated with a beneficial health-related QoL, particularly among the elderly with chronic diseases [19]. We also observed a negative correlation between NYHA and functional capacity in our study, indicating that as the severity of symptoms increases, as determined by the NYHA classification, there is a decrease in the ability to perform daily activities. Prior research supports this conclusion, indicating that the severity of symptoms is the primary predictor of functional status in patients with HF [20].

According to our research evidence, the female group showed significantly greater participation in HIIT compared to the male group. This finding is consistent with studies showing that women are more likely to improve their performance with shorter HIIT recovery intervals, suggesting the need for sex-specific exercise strategies [21]. Our findings indicate that women have higher scores concerning QoL when compared to men. Yet, another study showed that there were no statistically significant gender variations in the aspect of perceived QoL, highlighting the even more important role of age in subjective well-being. Such a contrast leads to the assumption that context or other population differences may impact the perceived QoL across diverse genders [22]. Male patients in our study reported more severe symptoms, as indicated by a higher NYHA class. This, however, varies with available literature that suggests a more symptomatic profile in female patients. This discrepancy is likely indicative of variation in the fundamental cardiac pathology, reporting pattern, or cohort specialization [23]. Our results indicate that women reported higher self-reported functional capacity; however, another competitive study using objective physical performance measures recorded better results for men in both strength and body mass. This distinction could represent the disparity between the perceptions and measured physical capacity [24].

We observed a substantial improvement in KCCQ-12 over time, indicating that our HF patients experienced an improvement in their self-reported health status. It suggests that participants experienced improved symptom control and QoL over time. These results were also observed in the earlier study in which a self-care intervention produced clinically significant changes (improvements) in KCCQ-12 scores of patients. Such findings can be used to validate the use of KCCQ-12 to monitor the positive aspects of well-being change over time in patients [25]. In our analysis, the NYHA functional class increased between T1 and T3, indicating a rise in reported symptom severity over time, despite improvements in self-reported functional capacity and QoL (KCCQ and DASI). This aligns with previous findings, where some patients experienced worsening NYHA class within one year, highlighting the importance of monitoring symptom progression [26]. The findings of our study indicated substantial enhancements in physical capability, as demonstrated by the elevated DASI scores over time. This corresponds to a prior review that indicated HIIT successfully boosts functional capacity in patients with HF, advocating the importance of structured exercise in rehabilitation [27].

In our research, KCCQ scores increased significantly over time, indicating an improvement in QoL and a decrease in symptom burden in patients with HF. In much the same way, another study found that patients with improving KCCQ-12 trajectories following discharge experienced improved health outcomes and lower mortality, once again demonstrating the clinical value of monitoring QoL changes over time [28]. In our study, there was a decline in the symptom status as NYHA class scores worsened over time, particularly those with a history of long-standing HF. This is in line with the results of a big registry-based study, which also indicated that the deterioration in NYHA class is related to the longer duration of HF and predicts worse clinical outcomes [26]. In our research, a significant improvement in DASI scores over time was observed, indicating an improvement in the cardiac function of subjects with HF. This observation can be explained by previous studies that indicate DASI measurements correspond to peak oxygen consumption results and that DASI can be used to differentiate between low- and high-risk patients based on their physical capabilities [29].

We found that the frequency of HIIT was significant and improved the QoL for patients with HF. This finding is consistent with earlier reports of similar effects on physical and mental health in adults who rarely exercise [15]. In our study, older age was a predictor of worse QoL improvement. This aligns with past findings, which have emphasized that the factors affecting QoL in older adults are complex and typically narrow, with factors such as health, social relations, and services being cited at the top of the list [30]. We found that the improvement in QoL was greater in women over time. This, however, differs from the previous findings, in which no significant gender differences in life satisfaction had been documented; thus, the effects of gender on QoL may vary across different populations or study contexts [22]. We have found that the type of HF was a good predictor of improvement in the QoL, which is consistent with meta-analytic evidence that although exercise training positively affects KCCQ scores in both HFpEF and HFrEF patients, the magnitude and nature of changes differ significantly, with HFrEF patients more likely to achieve a broader range of physiological changes [31]. Our results align with the available evidence, with a recent meta-analysis showing that comorbidities among HF patients are strongly correlated with a worse health-related QoL and prognosis, and comorbidity impedes optimal HF management [32].

We found that higher levels of participation in HIIT were strongly associated with lower NYHA class, which reflects a lower symptom burden. This finding aligns with earlier studies that have demonstrated HIIT improves functional ability and reduces the severity of symptoms in patients with HF [16]. In our study, the improvement in functional status (NYHA class) was higher in younger patients, which is consistent with previous studies that have shown older age is associated with lower physical performance and functional limitations [33]. In our study, women improved their NYHA class more than men. Although a more serious symptom burden is found in women, as reported previously, this could rationalize the larger improvement potential observed in our results [23]. We found that some subtypes of HF had a reduced likelihood of improving NYHA class. This is in line with findings that have shown that the patients facing the risk of T2DM-related HF had lower exercise capacity and subclinical cardiac dysfunction, suggesting a slower functional recovery of these patients [34]. In our study, the severity of comorbidities was associated with a worse rate of NYHA class improvement, a finding confirmed by other recent trials that revealed an adverse impact of greater comorbidities on health status and physical functioning among patients with HFrEF and HFpEF [35].

Our results, indicating that increased HIIT involvement is associated with higher exercise capacity, are corroborated by previous findings revealing better increases in VO_2_ peak and walking distance after HIIT intervention compared to moderate continuous training [17]. In agreement with earlier findings regarding the connection between age and physical deterioration, our results indicated that younger patients experienced greater physical functional improvement following the intervention compared to older patients, consistent with findings that slower physical recovery and higher age-related decline are associated with older age [36]. In our study, women showed higher improvements in the physical performance scores than men. In contrast, previous studies have indicated that, due to their higher baseline muscle mass and strength, men tend to make more changes to their physical performance. Thus, other factors may be present in addition to muscle mass that affect outcomes of exercise, including baseline levels of activity or responsiveness to rehabilitation [24]. We have found that the subtype of HF plays a significant role in determining the improvement in exercise capacity after HIIT, as some subtypes are more responsive to HIIT. This observation aligns with previous studies indicating that the HFpEF group improved the VO_2_ peak more than the HFrEF group following endurance training sessions [37]. Our observation that greater comorbidity burden was predictive of more minor improvements in exercise ability aligns with recent data demonstrating that comorbidity burden increasingly lowers peak VO_2_, walking, and QoL in patients with HF. This suggests that the benefits of exercise-based interventions may be severely limited by the presence and type of comorbidities [38].

Patients with HFrEF in our study were more likely to be diagnosed with the disease within six months compared to those with HFpEF, who had a longer diagnosis period. This finding is consistent with existing studies, which have shown that HFpEF is often underrecognized or diagnosed late due to difficulties in classifying its subtypes [39]. Our finding that HFpEF patients present a greater burden and broader spectrum of comorbidities aligns with recent systems medicine studies, which have revealed that HFpEF is associated with complex and varied clusters of comorbidities, particularly metabolic and inflammatory conditions [40].

Limitations

The present study has several limitations. First, the final sample size (N = 158) was smaller than the initially calculated sample of 300. Although adequate for detecting within-subject changes, this reduced sample size decreases the statistical power and limits the generalizability of our findings, especially across more diverse populations. Second, a self-created HIIT participation questionnaire was used, which was developed solely to screen participants. Neither was the tool officially validated, nor did the instrument possess psychometric properties such as reliability or construct validity, which could influence the accuracy of the measured participation levels. Also, as HIIT participation was assessed only through self-report and not by objective monitoring, the exact exercise protocol could not be replicated. Third, all outcome measures were self-reported, and none of the instruments were available in the local language, which increased the risk of response bias in the research. Fourth, the observational nature of the study does not allow us to conclude that the observed patterns are causal, and the lack of a comparison group (e.g., moderate-intensity training or standard care) limits the number of direct comparisons that can be made. The fourth significant limitation is the absence of a control group, which makes it difficult to attribute improvements solely to HIIT. The observed benefits may partly reflect the effects of concurrent medical treatment or the natural progression of the disease. Randomized controlled trials are needed to confirm causality. Another limitation is the reliance on self-reported outcome measures (KCCQ-12, DASI, and NYHA), which introduces the potential for response bias. Furthermore, the lack of objective physiological indicators, such as VO₂ max or echocardiographic parameters, restricts the ability to confirm whether the observed improvements reflect actual changes in cardiac function. Finally, the research only covered one city and a relatively limited healthcare facility, which may restrict the generalizability of the findings to other regions or more diverse clinical settings. Additionally, the short follow-up period limits the assessment of long-term outcomes, including mortality. Future studies with longer follow-up periods are warranted.

Future directions

Future studies may focus on randomized controlled trials comparing HIIT with other forms of exercise, such as MICT, to identify comparative efficacy. Additionally, new research involving a larger, more diverse sample and multiyear follow-ups may provide stronger findings on how people fare in the long term, including mortality rates and the frequency of repeated hospitalizations. As the current study employed a self-structured HIIT participation questionnaire, which was developed based on screening rather than psychometric validation, further research is needed to create and psychometrically validate standard tools for HIIT engagement in clinical populations. All assessment tools should also be translated and culturally adapted into the local languages to enhance inclusiveness and the accuracy of data. Future studies should employ randomized controlled designs that directly compare HIIT with MICT or standard care to strengthen causal inference. Additionally, psychosocial and behavioral factors that influence exercise adherence, particularly among different age groups and genders, may be investigated to implement HIIT-based cardiac rehabilitation more sustainably and in a more individualized manner.

## Conclusions

In this paper, we conclude that long-term HIIT is associated with a marked improvement in functional status, symptom burden, and QoL in patients with HF. These advantages are more prominent among younger individuals, women, those recently diagnosed, and those with fewer comorbidities. Although the observational design does not permit conclusive causation, the results confirm the clinical usefulness of incorporating HIIT into HF rehabilitation programs periodically, as well as the necessity of early, individualized intervention strategies to achieve optimal patient-centered results.

## Appendices

**Table 11:** High-intensity interval training (HIIT) participation

Items	Responses
On average, how many HIIT sessions do you perform per week?	-
On average, how long is each HIIT session?	-
For how many months have you been regularly doing HIIT?	-
How would you rate the intensity of your HIIT workouts?	-
